# Impact of Airway Inflammation on the Efficacy of CFTR Modulators

**DOI:** 10.3390/cells10113260

**Published:** 2021-11-22

**Authors:** Carla M. P. Ribeiro, Martina Gentzsch

**Affiliations:** 1 Marsico Lung Institute and Cystic Fibrosis Research Center, School of Medicine, University of North Carolina, Chapel Hill, NC 27599, USA; 2 Division of Pulmonary Diseases, Department of Medicine, School of Medicine, University of North Carolina, Chapel Hill, NC 27599, USA; 3 Department of Cell Biology and Physiology, School of Medicine, University of North Carolina, Chapel Hill, NC 27599, USA; 4 Division of Pediatric Pulmonology, Department of Pediatrics, School of Medicine, University of North Carolina, Chapel Hill, NC 27599, USA

**Keywords:** cystic fibrosis, CFTR, airway inflammation, CFTR corrector, CFTR potentiator, F508del rescue, primary bronchial epithelia

## Abstract

Defective CFTR biogenesis and activity in cystic fibrosis airways leads to airway dehydration and impaired mucociliary clearance, resulting in chronic airway infection and inflammation. Most cystic fibrosis patients have at least one copy of the F508del CFTR mutation, which results in a protein retained in the endoplasmic reticulum and degraded by the proteosomal pathway. CFTR modulators, e.g., correctors, promote the transfer of F508del to the apical membrane, while potentiators increase CFTR activity. Corrector and potentiator double therapies modestly improve lung function, whereas triple therapies with two correctors and one potentiator indicate improved outcomes. Enhanced F508del rescue by CFTR modulators is achieved by exposing F508del/F508del primary cultures of human bronchial epithelia to relevant inflammatory stimuli, i.e., supernatant from mucopurulent material or bronchoalveolar lavage fluid from human cystic fibrosis airways. Inflammation enhances the biochemical and functional rescue of F508del by double or triple CFTR modulator therapy and overcomes abrogation of CFTR correction by chronic VX-770 treatment in vitro. Furthermore, the impact of inflammation on clinical outcomes linked to CFTR rescue has been recently suggested. This review discusses these data and possible mechanisms for airway inflammation-enhanced F508del rescue. Expanding the understanding of how airway inflammation improves CFTR rescue may benefit cystic fibrosis patients.

## 1. Introduction

Cystic fibrosis (CF) lung disease results from a series of functional changes due to mutations in the CF transmembrane conductance regulator (CFTR). Alterations or the absence of CFTR function in the epithelia lining the airways causes decreased Cl^−^ secretion associated with increased Na^+^ reabsorption [1,2]. As a result, CF patients suffer from airway dehydration [3], accumulation of thickened mucus, and impaired mucociliary clearance [4,5,6]. These changes are coupled with persistent bacterial infections and chronic inflammation [7,8,9,10,11,12,13,14,15], which are detrimental to CF airways [16].

Current therapies combining CFTR modulators aim to improve CFTR activity in CF patients with the most common mutation (F508del CFTR) or other responsive mutations. Notably, little is known regarding the impact of the CF airway inflammatory environment on the activity of CFTR modulators; hence, studies are needed to understand the relationship between the levels of airway epithelial inflammation and the efficacy of CFTR modulators.

In this review, we summarize the present knowledge of the influence of airway inflammation regarding the efficacy of modulator-mediated rescue of CFTR in airway epithelia. The findings indicate that airway inflammation enhances CFTR rescue by current modulators in vitro and in vivo, and justify further research addressing the mechanisms mediating inflammation-increased CFTR rescue. An understanding of the interplay between anti-inflammatory treatments and CFTR modulator drugs may lead to new strategies for improving F508del activity in response to CFTR modulator therapy and benefit CF patients.

## 2. Cystic Fibrosis Airway Epithelia Are Inflamed

Earlier studies have indicated that the inflammatory response of CF airways is “excessive” [17,18]. CF airway epithelia are inflamed and display activation of nuclear factor-κB (NF-κB), increased production of pro-inflammatory cytokines, and reduced secretion of anti-inflammatory mediators [19,20,21,22]. By secreting inflammatory mediators in response to the infectious/inflammatory milieu of CF airways, CF airway epithelia contribute to the chronic inflammatory status of CF airways [23,24,25].

Our previous studies have shown that the endoplasmic reticulum (ER) Ca^2+^ stores are expanded in inflamed airway epithelia [26] and amplify Ca^2+^-mediated cytokine production [19]. This adaptive response should be beneficial for normal airways undergoing acute infection, since healthy airways are competent to clear the infectious insult. However, in obstructed CF airways, the amplified inflammatory responses resulting from airway epithelial ER Ca^2+^ store expansion are likely ineffective for clearing chronic infections in the presence of thickened mucus; thus, the inability to clear infections should contribute to damaging the airway walls [19].

Airway epithelial inflammation also increases the expression levels of ER chaperone proteins, e.g., calreticulin, GRP78/BIP, PDI [19,26,27]. Together with the expansion of the ER compartment and its Ca^2+^ storage, the up-regulation of ER chaperone proteins serves to increase the ER protein folding capacity, which is a necessary cellular function to accommodate the increased production of inflammatory mediators by inflamed airway epithelia. As discussed below, the increased ER protein folding capacity in inflamed CF airway epithelia likely contributes to enhance the efficacy of CFTR modulators.

## 3. In Vitro Translational Models of CF Airway Epithelial Inflammation

Several years ago, we developed a translational model of CF airway epithelial inflammation, which has proven very useful for studying many aspects of CF airway epithelial pathophysiology related to inflammatory responses. This model consists of mucosally exposing well-differentiated primary cultures of F508del/F508del human bronchial epithelia (HBE), grown at the air–liquid interface, to supernatant from mucopurulent material (SMM) obtained from the airways of excised human CF lungs [19,26,27,28,29,30]. SMM represents the “infectious and inflammatory soup” present in CF airways and contains products from bacteria, factors derived from neutrophils, including neutrophil elastase, MMP9, cathepsin G, BPI, and lysozyme, cytokines secreted from macrophages and airway epithelia [29], mucins, hundreds of peptides, and purines [31]; its cytokine composition is reproducible from patient to patient [29].

As SMM is obtained from CF lungs with severe disease, an additional model was developed where bronchoalveolar fluid (BALF) from pediatric CF patients with less severe disease was used in lieu of SMM [32]. Similar to the approach with SMM, BALF from CF patients contains the infectious and inflammatory factors to which the airway epithelia from pediatric patients are exposed. As CF airway epithelia are simultaneously exposed to all factors present in SMM or BALF in vivo, the use of SMM and BALF provides a superior experimental approach vs. the use of single inflammatory components for evaluating the therapeutic efficacy of CFTR modulators under inflammatory conditions relevant to CF airways, as detailed below.

## 4. Inflammation Enhances the Efficacy of CFTR Modulator Therapy

The development of CFTR modulators, such as correctors that augment F508del CFTR transfer to the apical membrane, and potentiators that increase CFTR channel activity, permitted successful treatment of the basic defect in CF [33]. The first FDA-approved CFTR modulator was the potentiator ivacaftor (VX-770), which improves the function of the gating mutant G551D CFTR [34,35]. While the potentiator VX-770 or the CFTR corrector lumacaftor (VX-809) alone did not significantly improve lung function in F508del CF patients [36], combining VX-809 with VX-770 (in the drug Orkambi) or combining the newer corrector tezacaftor (VX-661) with VX-770 (in the drug Symdeko) resulted in only modest lung function improvements in clinical trials in patients homozygous for F508del CFTR [37,38,39,40].

Recent triple combination treatment with the next generation correctors elexacaftor (VX-445) or bamocaftor (VX-659) resulted in significant improvement of clinical responses [41,42]. The drug Trikafta (a combination of VX-445, VX-661, and VX-770), which showed substantial efficacy in phase 3 clinical trials [43,44], was initially approved by the FDA for the treatment of CF patients who had at least one copy of the F508del mutation; subsequently, Trikafta was approved for other mutations.

The development of the above CFTR modulators is allowing the successful treatment of the basic CF defect. Several additional CFTR modulators have been developed during the last decade and are currently in different stages of the development pipeline; this topic has been addressed in recent publications [45,46,47,48]. The subsections below will review recent data on the impact of airway inflammation on CFTR rescue by modulators currently approved to be used in the clinic.

### 4.1. Evaluation of CFTR Rescue in Inflamed Airway Epithelia In Vitro

Previous studies have indicated that various cytokines, including IL-1β, IL-4, TNFα, IL-10, and IL-13, enhance CFTR activity [49,50,51,52,53]. In contrast, the inflammatory mediator TGFβ might have a negative impact on CFTR expression and F508del CFTR rescue. For instance, TGFβ has been shown to decrease the levels and function of apical CFTR in airway cells from non-CF subjects [54,55]. In addition, TGFβ has been reported to decrease CFTR biogenesis and inhibit the rescue of F508del CFTR [56]. A previous study has also indicated that a short exposure time (6 h) to a single bacterial stimulus, *Pseudomonas aeruginosa,* causes a reduction in Cl^−^ secretion by rescued F508del [57]. These studies indicate that individual inflammatory mediators can enhance or inhibit CFTR expression and function and, thus, can have different impacts on CFTR rescue.

**Figure 1:**
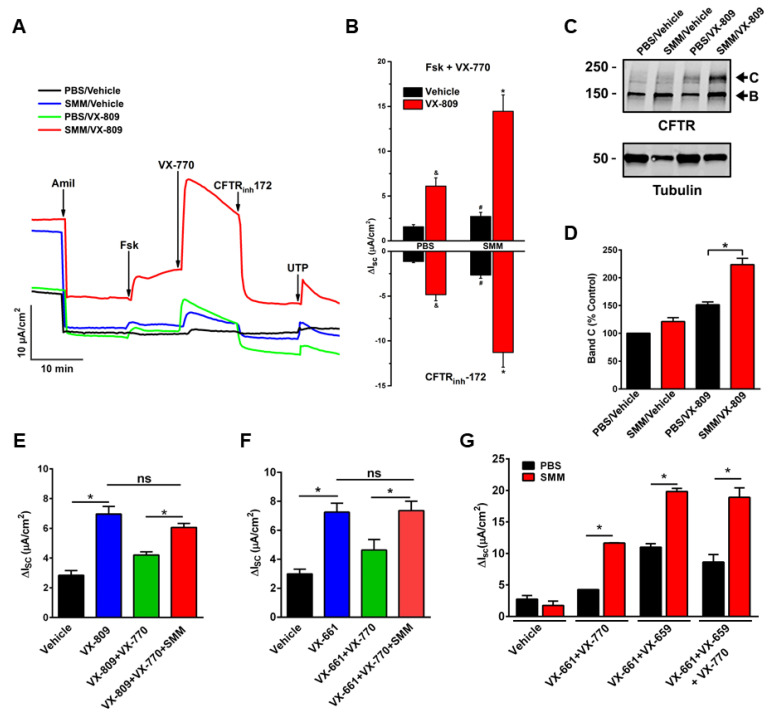
Exposure of CF HBE to SMM enhances F508del CFTR rescue. (**A**–**D**): CF HBE (F508del/F508del) cultures were exposed for 24 h to vehicle or 5 µM VX-809 in combination with 30 µL mucosal PBS or SMM. (**A**) Representative short-circuit currents (Isc, µA/cm^2^) recorded in Ussing chambers. (**B**) Quantification of F508del-mediated responses to forskolin (Fsk, 10 µM) + VX-770 (1 µM) and CFTRinh-172 (10 µM). (**C**) Representative Western blot of immunoprecipitated CFTR and tubulin controls. (**D**) Quantification of band **C** as % values normalized from band **C** values from control (vehicle- and PBS-treated CF HBE). (**E**) SMM overcomes chronic VX-770 treatment (5 μM, 48 h)-mediated abrogation of VX-809-promoted F508del CFTR rescue. (**F**) SMM overcomes chronic VX-770 treatment (5 μM, 48 h)-mediated abrogation of VX-661-dependent F508del rescue. (**G**) SMM enhances triple combination-promoted F508del CFTR rescue. CFTR responses to acute forskolin + VX-770 from F508del/F508del HBE in Ussing chambers. HBE were treated with VX-659 (1 µM), VX-661 (5 µM), and VX-770 (5 µM). All data are expressed as mean ± SEM. (**A**–**F**): *n* = 3–4 CF HBE donors, 3–4 cultures/donor. (**G**): 2 CF HBE donors, 5–6 cultures/donor. * *p* < 0.05. (**A**–**D**): Modified from Gentzsch et al., 2018 [58] (reproduced with permission of the ©ERS 2021: European Respiratory Journal 52 (6) 1801133; doi: 10.1183/13993003.01133-2018 Published 20 December 2018). (**E**–**G**): Modified from Gentzsch et al., 2021 [32].

**Figure 2:**
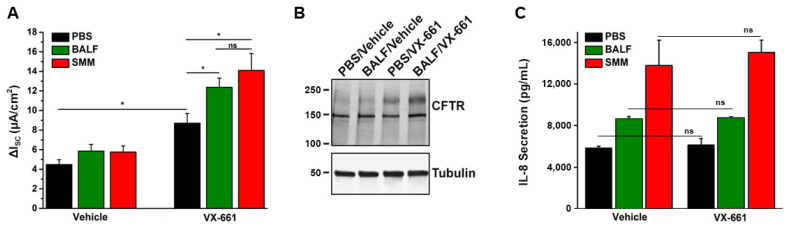
Exposure of CF HBE to pediatric BALF enhances F508del CFTR rescue. (**A**) CFTR responses from F508del/F508del HBE cultures evaluated in Ussing chambers. Cultures were apically exposed to 30 µM BALF or SMM and serosally treated with vehicle or 5 µM VX-661. Data are expressed as mean ± SEM. ns = not significant; 2 donors, five to six cultures/experimental group. (**B**) Representative CFTR Western blot to analyze F508del maturation (upper band); (**C**) IL-8 secretion (pg/mL of culture media) from the F508del/F508del HBE evaluated in (**A**). * *p* < 0.05. Data are expressed as mean ± SEM. Modified from Gentzsch et al., 2021 [32].

Until recently, no studies have been conducted to evaluate the efficacy of CFTR modulators in CF airway epithelia that have been inflamed by the native CF airway milieu containing various inflammatory mediators. To test the effect of CFTR modulators in in vitro translational models of inflammation relevant to CF airways, we utilized F508del/F508del primary cultures of CF HBE mucosally exposed to SMM [32,58] or BALF from pediatric CF patients [32]. Our first study indicated that SMM exposure potentiated F508del CFTR activity resulting from the treatment with the CFTR corrector VX-809 and the potentiator VX-770 (Figure 1A,B). SMM exposure also enhanced the formation of the mature (band C) F508del CFTR triggered by the treatment with the CFTR modulators (Figure 1C,D) [58]. These responses were independent of increases in CFTR mRNA [58]. In a subsequent study, we demonstrated that exposure of F508del CF HBE cultures to BALF from CF children enhanced CFTR activity (Figure 2A) and maturation (Figure 2B) resulting from treatment with the CFTR corrector VX-661 and the potentiator VX-770 [32], similar to the potentiating effect of SMM on CFTR rescue [58].

Strikingly, inflammation can also overcome the destabilization effect of chronic VX-770 exposure on CFTR rescue. Previously, we have established that chronic treatment with VX-770 abrogates corrector-mediated rescue of F508del by enhancing internalization and turnover of mature CFTR proteins [59]. Subsequently, we demonstrated that SMM exposure enhanced F508del CFTR responses to chronic treatment with VX-809 (Figure 1E) or VX-661 (Figure 1F) plus VX-770 by overcoming chronic VX-770-promoted inhibition of F508del rescue [32]. We have also shown that chronic VX-770 treatment inhibits UTP-induced activation of the calcium-activated chloride channel (CaCC) [59]. However, in follow-up studies, we demonstrated that the inhibitory effect of VX-770 on UTP-induced CaCC responses was reversed by exposing the F508del cultures to SMM [32]. Thus, whilst chronic VX-770 treatment can decrease F508del CFTR and CaCC activities, the inhibitory effect of VX-770 is overcome by airway epithelial inflammation.

Notably, the enhancing effect of SMM on CFTR rescue is also observed in CF HBE cultures treated with various combinations of modulators, including a triple combination (Figure 1G).

### 4.2. Airway Inflammation Enhances CFTR Modulator Therapy-Improved Lung Function In Vivo

The above studies demonstrated that the CF airway inflammatory milieu has a positive impact on the rescuing activity of CFTR modulators. In addition, as discussed earlier, various publications have reported an enhancement in CFTR function by certain cytokines in vitro [49,50,51,52,53]. However, little is known regarding whether airway inflammation affects the rescue of mutant CFTR in CF subjects.

Recently, Rehman et al. reported that inflammation enhances the response to CFTR modulators in vitro and in vivo [60]. The in vitro studies employed TNFα and IL-17 as inflammatory stimuli [60], and their findings are in agreement with our previous in vitro studies demonstrating that the CF airway inflammatory milieu enhances the efficacy of CFTR modulators [32,58]. As the stability of band C was not evaluated and band B was not detected in the study by Rehman et al. [60], the mechanism for the enhanced F508del CFTR rescue by TNFα and IL-17 remains unclear.

Importantly, Rehman et al. observed a positive correlation between airway inflammation and lung function improvement in CF patients treated with VX-770 [60]. As the majority of the G551D and R117H patients in the study by Rehman et al. had F508del in one allele [60], we speculate that maturation of F508del may have been increased by inflammation, contributing, at least in part, to the observed clinical improvement. This is supported by the observation that inflammation enhances the ER folding capacity [19,26,27,61], which may facilitate rescue of misfolded CFTR [32]. Furthermore, because we have demonstrated that R117H is also a folding mutant [62], it may exhibit improved folding in the presence of inflammatory stimuli. As discussed above, although VX-770 destabilizes rescued F508del [59,63], inflammation can overcome the detrimental effects of chronic exposure to ivacaftor [32]. Hence, we speculate that through a mechanism involving inflammation-dependent CFTR stabilization, the efficacy of chronic VX-770 treatment is improved in CF patients, resulting in increases in forced expiratory volume in 1 s (FEV_1_), as observed by Rehman et al. [60].

Additional studies are needed to examine the mechanism(s) responsible for the augmentation of CFTR rescue under inflammatory conditions. Addressing how inflammation enhances the efficacy of CFTR modulators might lead to novel therapies exploiting the beneficial effects of inflammation on CFTR rescue, while mitigating its harmful consequences to CF airways.

## 5. Do CFTR Modulators Decrease the Inflammatory Status of CF Airways?

With the emergence and access to highly effective CFTR modulator therapies, it is of broad interest for doctors, CF patients, and researchers to know whether these therapies that target the basic defect in CF also alleviate airway inflammation in CF airways.

### 5.1. The Impact of CFTR Modulators on CF Airway Epithelial Inflammatory Responses In Vitro

Previous studies tested the impact of VX-809 and VX-770 on *Pseudomonas aeruginosa*-triggered inflammatory responses in primary HBE cultures from F508del CF patients [64]. It was found that these CFTR modulators inhibited the up-regulation of the mRNA levels from the cytokines CXCL1, CXCL2, and CXCL8 (IL-8) resulting from *Pseudomonas aeruginosa* exposure [64], suggesting that VX-809 and VX-770 have anti-inflammatory properties.

Recently, we tested whether CFTR modulators exhibited anti-inflammatory effects in vitro, using the SMM translational model. Utilizing individual CFTR modulators or several combinations of CFTR correctors (VX-809, VX-661, or VX-659) and the potentiator VX-770, we have shown that CFTR rescue does not decrease the inflammatory status of homozygous F508del CFTR primary HBE cultures exposed to SMM [32]. In an additional study, this was also demonstrated for a triple combination of CFTR modulators currently used in the clinic by showing that SMM-increased IL-8 secretion was not altered by treatment with VX-445, VX-661, and VX-770 [30]. Figure 2C illustrates that pediatric BALF- or SMM-increased IL-8 secretion in F508del cultures is not affected by treatment with VX-661 [32]. Hence, these studies indicate that current CFTR modulators do not decrease the up-regulation of CF airway epithelial cytokine production resulting from exposure to the infectious/inflammatory CF airway milieu and, thus, do not exhibit anti-inflammatory properties. Our findings are in contrast with the studies of Ruffin et al. [64], and likely reflect the different modes to induce HBE inflammation, e.g., a single stimulus (*Pseudomonas aeruginosa*) vs. a holistic approach (SMM, BALF) relevant to the infectious and inflammatory milieu present in CF airways. For a detailed review on the impact of CFTR modulators on inflammatory responses, including the function of monocytes, please see reference [65].

Our data also suggest that ER retention of endogenous F508del CFTR is not pro-inflammatory per se in primary HBE cultures, since the functional CFTR rescue to the apical membrane did not decrease the inflammatory status of CF epithelia [30,32]. Rather, these data indicate that airway epithelial inflammation in CF is likely an indirect consequence of impaired mucociliary clearance with buildup of mucus and bacterial accumulation. The lack of correlation between F508del CFTR function and CF bronchial epithelial inflammation is in agreement with our previous studies [19].

### 5.2. CFTR Modulators and CF Airway Inflammation In Vivo

Although the above studies have indicated that CFTR modulators do not possess anti-inflammatory responses in vitro, it is necessary to evaluate whether they exhibit anti-inflammatory properties in CF subjects. The impact of 6 months of ivacaftor treatment on airway inflammation was evaluated in the G551D Observational (GOAL) study with patients with at least one G551D CFTR allele and a predicted FEV_1_ ≥ 40% [66]. Although there was an overall improvement in FEV_1_ and decreases in sweat chloride, the data indicated that ivacaftor had no effect on bacterial pathogens, or inflammatory markers in collected sputa samples [66]. In agreement with these findings, BAL samples from clinically stable CF infants and young children who had been started on ivacaftor were collected through the Irish and the Australian programs Study of Host Immunity and Early Lung Disease (SHIELD CF) and Australian Respiratory Early Surveillance Team (AREST CF) and analyzed for IL-8, total and differential cell counts, and NE activity [67]. Two sequential annual BAL samples were analyzed before starting ivacaftor and one sample after treatment initiation and compared with samples from a control group of F508del homozygous children not undergoing modulator therapy [67]. No significant differences were found for IL-8, absolute neutrophil count, and NE positivity before or after ivacaftor treatment, and ivacaftor therapy was not a predictor of inflammatory markers in BAL [67]. Moreover, no significant differences were found regarding the number of detected pathogens in the BAL samples between the groups [67]. These studies indicated that, in stable preschool children with CF, ivacaftor is not therapeutically beneficial for airway inflammation and infection.

In contrast, other clinical trials suggested that CFTR modulators exhibit anti-inflammatory effects in CF airways. For instance, earlier studies were conducted in twelve G551D subjects with chronic airway infections before and after ivacaftor treatment. Ivacaftor reduced the levels of IL-8, IL-1β, and neutrophil elastase (NE) in sputa from these patients during the first week of treatment, with a continued decline in the levels of these inflammatory markers over the course of two years [68]. However, the G551D CF subjects continued to have persistent *Pseudomonas aeruginosa* infections [68]. In a longitudinal cohort study with ten G551D CF patients, results from inflammatory mediators in nasal lavages indicated that the levels of IL-1β and IL-6 declined during 12 weeks after initiation of ivacaftor treatment, whereas IL-8 levels were decreased only up to 8 weeks of treatment [69]. Another recent study evaluated several clinical outcomes, including pro-inflammatory cytokines in 12 years and older F508del homozygous patients before and 8–16 weeks after initiation of therapy with lumacaftor and ivacaftor [70]. While the modulators blunted the levels of IL-1β, the concentrations of other inflammatory mediators, e.g., IL-6, IL-8, TNFα, and NE activity, remained unchanged after therapy with these CFTR modulators [70].

The data from the above clinical trials reveal inconsistencies regarding whether CFTR modulators exert anti-inflammatory effects in airways of CF patients, and suggest that additional studies will be necessary to address this issue.

## 6. Conclusions and Future Directions

Our in vitro studies strongly suggest that airway epithelial inflammation enhances the therapeutic efficacy of various combinations of CFTR modulators currently being used by CF patients. However, our data should not imply that the inflammatory status of CF airways should be ignored, for chronic airway inflammation is highly detrimental to CF patients. Nevertheless, these findings suggest that research into the mechanism responsible for inflammation-increased CFTR rescue by pharmacological agents might lead to an improved efficacy of CFTR modulators.

**Figure 3:**
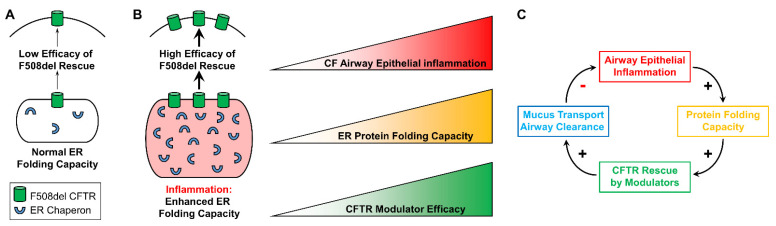
Proposed model for the interplay between airway epithelial inflammation and CFTR rescue. (**A**): In the absence of airway epithelial inflammation, the ER protein folding capacity is normal and associated with a lower efficacy of F508del recue. (**B**): Inflammation expands the ER and enhances its protein folding capacity, which should facilitate F508del CFTR rescue. We propose a linear correlation between airway epithelial inflammation, ER protein folding capacity, and efficacy of CFTR modulators in vitro. Modified from Gentzsch et al., 2021 [32]. (**C**): Proposed in vivo relationship between CF airway epithelial inflammation, ER protein folding capacity, CFTR rescue, and airway mucociliary clearance. Modified from Gentzsch et al., 2021 [32].

It can be speculated that inflammation-increased modulator-dependent CFTR rescue results, at least in part, from expansion of the ER protein folding capacity, which has been documented in inflamed CF airway epithelia [19,26,27,61]. Increases in protein folding resulting from up-regulation of ER chaperones should facilitate CFTR folding and trafficking in inflamed CF airway epithelia. Hence, we predict the existence of a positive relationship between the airway inflammatory status, the CF epithelial ER protein folding capacity, and the efficacy of CFTR modulators in the airways of CF subjects (Figure 3A,B).

Chronic treatment with VX-770 increases the internalization and turnover of mature CFTR protein, and counteracts corrector-mediated rescue of F508del [59,63]. It is possible that chronic VX-770 treatment alters the lipid bilayer properties because of its lipophilicity [71]. Notably, inflammation abrogates the negative effects of chronic VX-770 treatment on CF airway epithelial F508del CFTR responses [32]; this may result from the inflammation-inhibited destabilizing effect that chronic VX-770 treatment exerts on CFTR [32]. Future studies will need to address the mechanism of the protective effect of inflammation against VX-770-induced destabilization of CFTR, including whether inflammation alters the biochemical and biophysical properties of the apical membrane to increase CFTR stabilization.

The in vitro findings indicating that CF airway epithelial inflammation enhances the efficacy of CFTR modulators, together with the initially predicted [32] and the recently found positive correlation between airway inflammation and enhanced CFTR modulator-improved lung function in CF patients [60], have implications for the clinic. For instance, no study has addressed whether anti-inflammatory medications (e.g., azithromycin) interfere with the efficacy of CFTR modulators. Understanding the interplay between the levels of airway inflammation and CFTR modulator efficacy, as illustrated in Figure 3C, will likely lead to improvements of CFTR modulator therapies and further benefit CF patients. Triple CFTR modulator therapy may reduce the requirement for intravenous antibiotics treatment, and increasing the understanding of the efficacy of modulators in inflamed and infected CF airways may enable refinement of therapeutic regimen projections [72]. While Trikafta therapy is proving beneficial for most CF patients, there is still an unmet need to improve the efficacy of CFTR-targeting treatments for patients with rare mutations, whose mutant CFTR proteins are less responsive to available therapies.

Future studies are necessary to explore the role of possible pathways that can explain our proposed model for how inflammation enhances CFTR rescue. Detailed knowledge regarding the mechanisms by which inflammation enhances the rescue of F508del CFTR in response to CFTR modulators and up-regulates CFTR function may not only benefit CF patients but also give insights into novel therapies for other diseases where CFTR function is diminished.

## Data Availability

Not applicable.
